# La EFLM *Academy* – ventajas y oportunidades

**DOI:** 10.1515/almed-2021-0038

**Published:** 2021-05-18

**Authors:** Ana-Maria Simundic

**Affiliations:** Departamento de Diagnóstico de Laboratorio Médico, Hospital Universitario “Sveti Duh”, Sveti Duh 64, 10 000 Zagreb, Croacia; Facultad de Farmacia y Bioquímica, Universidad de Zagreb, Zagreb, Croacia; Presidenta de la EFLM, Milan, Italy; Directora del Grupo de trabajo del Curso Syllabus sobre el Programa Formativo de la EFLM, Milan, Italy; Miembro de la Junta Directiva de la IFCC (International Federation of Clinical Chemistry and Laboratory Medicine), Milan, Italy

## Historia de la EFLM

La Federación Europea de Química Clínica y Medicina de Laboratorio (EFLM) es una organización relativamente joven, aunque sus raíces se remontan a principios de los años setenta, cuando empezaron a manifestarse las primeras preocupaciones sobre la comparabilidad de la profesión entre los distintos países europeos y de ahí surgió la idea de regular la profesión. Los primeros años de andadura de la EFLM quedaron bien documentados por el personal de la EFLM, y este documento, aunque nunca ha sido publicado oficialmente en ninguna revista, es la única historia escrita de la EFLM [1]. La EFLM se fundó oficialmente en 2007 con la fusión del Foro de Sociedades Europeas de Química Clínica (FESCC) y la Confederación de Comunidades Europeas de Química Clínica (EC4) en la Asamblea General de Euromedlab, celebrada en Amsterdam. Inicialmente se le dio el nombre de Federación Europea de Química Clínica (EFCC), pero este fue modificado en 2013 para añadir “y de Medicina de Laboratorio”, para reflejar el carácter global de la EFLM como la principal organización de medicina de laboratorio de Europa.

La fusión del FESCC y la EC4 creó una verdadera sinergia, al haber aunado en la EFLM los valores, competencias y actividades de las dos organizaciones. Mientras que la actividad del FESCC esencialmente se centraba en la promoción de la educación y el avance científico en la profesión, la misión principal de la EC4 consistía en regular la profesión en Europa. Uno de los objetivos estratégicos clave de la EC4 era lograr una Directiva Sectorial para los especialistas en química clínica comparable a la Directiva Médica, y estaba claro que el camino para lograr dicho objetivo pasaba por elaborar un inventario de la formación teórica y práctica ofrecida en distintos países de la UE en aquel momento. A su vez, se esperaba que, a cambio de dichos esfuerzos, se abriera la posibilidad de crear un registro de especialistas en química clínica de toda Europa (el Registro). Esto desembocó en la publicación de la primera versión del Programa Europeo de Formación de Posgrado en Química Clínica y sus sucesivas ediciones, siendo la última versión la de 2018 [2], [3], [4], [5]. Mientras que el objetivo del Programa era definir el ámbito formativo en química clínica y aquellas áreas en las que un especialista en química clínica debería ser capaz de mostrar sus conocimientos, competencias y capacitación, el propósito del Registro era garantizar la libertad de movimientos en la Unión Europea, estableciendo unos requisitos mínimos formativos y profesionales, así como competencias de gestión [6], [7], [8].

Desde su creación, la EFLM ha dado continuidad al camino marcado por sus predecesores. Aunque aún no se ha alcanzado su objetivo último, se podría decir que, posiblemente, estamos más cerca que nunca. Una de las actividades clave de la EFLM en los últimos 14 años (desde 2007 hasta la actualidad) ha sido completar el Registro y conseguir el reconocimiento de los especialistas en Medicina de Laboratorio. Gracias a dichos esfuerzos, el número de miembros del Registro ha aumentado sustancialmente, aunque la verdadera expansión se produjo con la creación de la EFLM *Academy* en enero de 2020.

## La EFLM *Academy*

La EFLM es una asociación de las sociedades nacionales de medicina de laboratorio de más de 40 países europeos, lo que representa más de 22.000 especialistas europeos en medicina de laboratorio. Los objetivos de la EFLM son los siguientes:

– Promover y mejorar la ciencia y educación en el campo de la química clínica y la medicina de laboratorio.
– Mejorar la eficiencia, calidad y seguridad del paciente, gracias a proporcionar una calidad óptima de los servicios de medicina de laboratorio.
– Representar a la especialidad de química clínica y medicina de laboratorio en los foros europeos, vis-à-vis ante los organismos políticos, profesionales y científicos, entre otros, incluyendo a las organizaciones de pacientes.
– Representar los intereses profesionales de los especialistas europeos en química clínica y medicina de laboratorio.
– Promover y mejorar la profesión de especialista en química clínica y medicina de laboratorio.
– Promover la certificación (¿) y registro de especialistas en química clínica y medicina de laboratorio a través del Registro de Especialistas Europeos en Química Clínica y Medicina de Laboratorio de la EFLM.

Para alcanzar sus objetivos y ayudar a ampliar el Registro de Especialistas Europeos en Química Clínica y Medicina de Laboratorio, la EFLM ha creado la EFLM *Academy*. La EFLM *Academy*, creada oficialmente el 1 de enero de 2020, es una plataforma de recursos y comunicación única, exclusivamente disponible on-line (en línea), a través de la cual la EFLM pretende fomentar la formación y capacitación, así como el desarrollo profesional continuo de los especialistas europeos en medicina de laboratorio. Como entidad profesional líder en medicina de laboratorio en Europa, el objetivo de la EFLM *Academy* es servir como recurso educativo no solo para los especialistas en medicina de laboratorio, sino también para todos aquellos interesados en la medicina de laboratorio.

## ¿Quién puede ser miembro de la EFLM *Academy*?

Se puede ser miembro de la EFLM *Academy* a título individual, pudiendo ser miembros todas aquellas personas interesadas en la medicina de laboratorio. También se admite a representantes de la industria de tecnologías de diagnóstico *in vitro*.

## ¿Cómo puedo yo unirme a la EFLM *Academy*?

Existen dos formas de inscribirse como miembro de la EFLM *Academy*: a) mediante una inscripción en bloque, centralizada a través de las respectivas Sociedades Nacionales, o b) mediante inscripción individual. El método más ventajoso es la inscripción en bloque, no solo porque esta forma es más cómoda para los socios de las sociedades nacionales, sino también porque ofrece más beneficios que la inscripción individual.

La inscripción en bloque se puede realizar en dos sencillos pasos:

1. Mediante acuerdo entre la EFLM y la Sociedad Nacional correspondiente.

La inscripción en bloque se realiza mediante un acuerdo suscrito por la EFLM y la Sociedad Nacional pertinente. Las sociedades nacionales interesadas en inscribirse a la EFLM *Academy* mediante inscripción en bloque pueden contactar con la Secretaría de la EFLM (eflm@eflm.eu) para recibir la propuesta de acuerdo. Cabe señalar que, una vez firmado un acuerdo, la inscripción de los profesionales adquirirá validez a inicios del siguiente año (esto es, si se firma un acuerdo en junio de 2021, la inscripción se activará el 1 de enero de 2022).

(2) Listado de miembros que se inscribirán en la EFLM *Academy*.

Una vez firmado el acuerdo entre la EFLM y la Sociedad Nacional correspondiente, la EFLM pedirá a dicha Sociedad que remita a la Secretaría de la EFLM un listado de los profesionales que pasarán a ser miembros de la EFLM *Academy* en el año siguiente. Esta lista deberá remitirse a finales de año, el 31 de diciembre como muy tarde.

La duración de la inscripción se extenderá desde el 1 de enero al 31 de diciembre. Dado que el citado procedimiento puede llevar un par de meses, se recomienda iniciarlo en la primera mitad del año, para poder contar con el tiempo suficiente a fin de completar el proceso.

La inscripción individual es una alternativa para los miembros de aquellas sociedades que, por alguna razón, no desean optar por la inscripción en bloque. Se pueden remitir las solicitudes en línea a la dirección: https://www.eflm.eu/academy-register/login. Tal como hemos mencionado anteriormente, aquellos miembros que se hayan inscrito a través de la inscripción individual no podrán disfrutar del paquete completo de ventajas de la inscripción centralizada.

## La EFLM *Academy* y el Registro

Mientras que la EFLM *Academy* está abierta a todas aquellas personas interesadas en la medicina de laboratorio, aquellos que también cumplan los requisitos de Equivalencia de Estándares (EoS, por sus siglas en inglés) de educación y capacitación pueden inscribirse en el Registro EuSpLM. A aquellos interesados en inscribirse en el Registro (ya sea mediante inscripción individual o centralizada) se les pedirá que acrediten que cumplen los requisitos de EoS. En el caso de las inscripciones centralizadas, dicha acreditación la proporcionará la Sociedad Nacional correspondiente. En el caso de las inscripciones individuales, será la persona solicitante la encargada de remitir la documentación pertinente. Para más información sobre el Registro puede visitar la página web de la EFLM, y buscar el Registro de Especialistas en Medicina de Laboratorio de la EFLM (EuSpLM) (https://www.eflm.eu/site/page/a/1305).

Las solicitudes de inscripción en el Registro serán evaluadas por el Profession Committee de la EFLM (C-P) y, aquellos solicitantes que cumplan los requisitos quedarán automáticamente inscritos en el Registro de la EFLM y será reconocido como EuSpLM.

**Figura 1:**
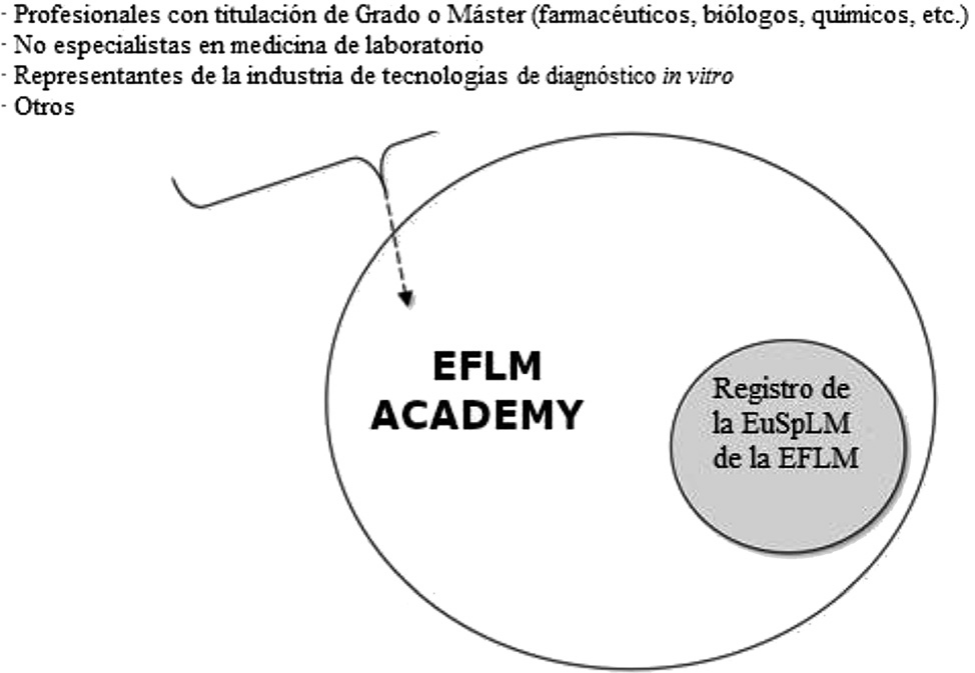
Relación entre la EFLM *Academy* y el Registro de la EFLM.

La inscripción en el Registro únicamente se puede realizar previa solicitud a la EFLM *Academy*. La relación entre el Registro y la EFLM *Academy* está descrita en la Figura 1.

## Ventajas para los miembros de la EFLM *Academy*

El paquete de beneficios de la *EFLM Academy* ha sido específicamente diseñado para satisfacer las necesidades de los profesionales de medicina de laboratorio en Europa. A continuación, se enumeran las ventajas de los miembros de la EFLM *Academy* vigentes en 2021:

– Inscripción gratuita a la versión online del CCLM, la revista oficial de la EFLM;
– Acceso libre y gratuito a los documentos del Clinical Laboratory Standard Institute (CLSI) (esto es únicamente aplicable a los miembros de la EFLM *Academy* con inscripción en bloque a través de su Sociedad Nacional);
– Reducción en la cuota de miembro del CLSI (descuento del 25%), aplicable a los miembros de la EFLM *Academy* con inscripción individual;
– Reducción en la cuota de inscripción en todos los cursos y conferencias de la EFLM (aplicable a las Conferencias Preanalíticas de la EFLM, las Conferencias Estratégicas de la EFLM, y los simposios de CELME. Esto no es aplicable a aquellos congresos o conferencias organizadas en colaboración con otras organizaciones como EuroMedLab),
– Acceso gratuito a los seminarios web de la EFLM;
– Notificaciones periódicas por correo electrónico con todas las actividades, programas y oportunidades de la EFLM.

Además, solo para miembros de las Sociedades o Asociaciones Nacionales de la EFLM:

– Opción de solicitar a la EFLM ayudas de desplazamiento;
– Inscripción en el Registro EuSpLM de aquellos que cumplan los requisitos de Equivalencia de Estándares de educación y capacitación de la EFLM.

La EFLM se compromete a ampliar las ventajas y seguir ofreciendo nuevas oportunidades a sus miembros en el futuro. Aunque dichas ventajas ya están disponibles en 2021, cabe mencionar que la Junta Directiva de la EFLM recientemente decidió aumentarlas sustancialmente, ofreciendo nuevas y excitantes oportunidades, tales como el acceso gratuito al Curso del Programa Formativo de la EFLM (*Syllabus*), el único recurso formativo en línea que abarca todas las actividades de medicina de laboratorio (disponible a partir de enero de 2022), la suscripción gratuita en versión online a otras revistas de medicina de laboratorio, una mayor oferta de formación en línea, y la oportunidad de participar en nuevos premios (2022), entre otras ventajas.

Esperamos que la EFLM *Academy* continúe creciendo en el futuro. Su valor no solo se demuestra por ofrecer apoyo formativo, sino también por ser un catalizador en el reconocimiento de los especialistas europeos de medicina de laboratorio.

El principal propósito de ser miembro de la EFLM *Academy*, más allá de los beneficios que ello supone, es contribuir a la armonización de la formación en medicina de laboratorio, así como apoyar a la EFLM en su lucha por conseguir el reconocimiento de los especialistas europeos en medicina de laboratorio.

Ser miembro no es solo una cuestión de elección, también se puede ver como una responsabilidad profesional. Invitamos a todos los profesionales a que se unan, ya que unidos somos más fuertes.
